# A qualitative assessment of the challenges of WHO prequalification for anti-malarial drugs in China

**DOI:** 10.1186/s12936-018-2303-8

**Published:** 2018-04-03

**Authors:** Yangmu Huang, Ke Pan, Danlu Peng, Andy Stergachis

**Affiliations:** 1 School of Public Health, Peking University, Xueyuan Road 38, Haidian District, Beijing, 100191 China; 2 School of Public Health, University of Washington, Seattle, WA USA; 3 School of Pharmacy, University of Washington, Seattle, WA USA

**Keywords:** Malaria, WHO prequalification, Anti-malarial, Artemisinins

## Abstract

**Background:**

While China is a major manufacturer of artemisinin and its derivatives, it lags as a global leader in terms of the total export value of anti-malarial drugs as finished pharmaceutical products ready for marketing and use by patients. This may be due to the limited number of World Health Organization (WHO) prequalified anti-malarial drugs from China. Understanding the reasons for the slow progress of WHO prequalification (PQ) in China can help improve the current situation and may lead to greater efforts in malaria eradication by Chinese manufacturers.

**Methods:**

In-depth interviews were conducted in China between November 2014 and December 2016. A total of 26 key informants from central government agencies, pharmaceutical companies, universities, and research institutes were interviewed, all of which had current or previous experience overseeing or implementing anti-malarial research and development in China.

**Results:**

Chinese anti-malarial drugs that lack WHO PQ are mainly exported for use in the African private market. High upfront costs with unpredictable benefits, as well as limited information and limited technical support on WHO PQ, were reported as the main barriers to obtain WHO PQ for anti-malarial drugs by respondents from Chinese pharmaceutical companies. Potential incentives identified by respondents included tax relief, human resource training and consultation, as well as other incentives related to drug approval, such as China’s Fast Track Channel.

**Conclusions:**

Government support, as well as innovative incentives and collaboration mechanisms are needed for further adoption of WHO PQ for anti-malarial drugs in China.

## Background

Access to affordable, safe, quality medicines is a key to achieving the Sustainable Development Goal (SDG) 3, namely, to ensure healthy lives and promote well-being for all at all ages. Indicators for SDG 3 include tracking the incidence of major infectious diseases, such as malaria and HIV [1]. To assure that active pharmaceutical ingredients (APIs) and finished pharmaceutical products (FPPs) for these and other priority diseases are safe, appropriate and meet stringent quality standards, the World Health Organization (WHO) established the Prequalification of Medicines (PQM) programme.

WHO prequalification (PQ) aims to provide qualified, safe and efficacious medicines for United Nations’ (UN) procurement agencies and to countries for bulk purchasing and distributing medicines in resource-limited countries [2]. In addition to UN agencies, other donors and agencies, such as the global fund to fight AIDS, tuberculosis and malaria (GF), have followed the UN’s example and also require WHO PQ for bulk purchasing of medicines. Since these large purchasers require WHO PQ, this designation can be a significant advantage for a company to receive WHO prequalified status for its FPP [3]. Currently, over 500 medicines have achieved WHO PQ status [4]. Approximately 70% of WHO PQ medicines are generic drug products [5].

China has been engaged in global health since the 1950s, having transformed from an aid-recipient country to an aid-donor country [6]. China’s foreign assistance to low-income countries includes dispatching medical teams, constructing health care delivery facilities, training health care personnel, and donation of drugs and medical equipment [7]. China is also a major manufacturer of the anti-malarial artemisinin (qinghaosu) and its derivatives, with an integrated industry encompassing *Artemisia annua* planting, exaction, research and development (R&D), drug production, and commercialization [8]. However, China is not the top sales country in terms of total export value of FPPs of anti-malarial drugs. The top ten countries of total export value of anti-malarial drugs are located in Asia (India, Pakistan), North America (USA), Africa (Sudan, Uganda, Ivory Coast, Nigeria), and Europe (Italy, The Netherlands, Switzerland) [9]. Certain European and Asian countries, including Switzerland, France and India, are major importers of China’s anti-malarial drugs. Since the burden of malaria is not endemic in Switzerland and France and is in the control phase in India, anti-malarial drugs exported to these countries are usually transported or processed for further sale in other countries where malaria is endemic.

The sale of anti-malarial drugs through PQ pharmaceutical companies in a third country not only limits profit for Chinese pharmaceutical companies, but also raises procurement cost and elapsed time from manufacturer to end users [9]. In order to provide affordable anti-malarial drugs to malaria-endemic countries, China could increase the amount of direct exportation of anti-malarial drugs. However, Chinese companies primarily rely on providing APIs for foreign companies to produce final anti-malarial drugs, while the direct exportation of anti-malarial drugs from China still faces the challenge of indirect sale due to the lack of having obtained WHO PQ certification [9]. While WHO and the Chinese Government have been engaged in WHO PQ procedures for over a decade, only 11 anti-malarial drugs produced by one pharmaceutical factory in China have achieved WHO PQ to date [4]. This study aims to examine the reason for the slow progress of WHO PQ in China, and discusses possible approaches to improve the situation of the dearth of WHO PQ anti-malarial drugs in China.

## Methods

### Study design

The study used qualitative research methods: in-depth interviews with key informants (KIs) located in China. A triangulation method was used to collect data. Qualitative data collection through interviews between November 2014 and June 2016 was conducted in the work place of KIs followed by on-site observations. Reporting of qualitative study methods follows the consolidated criteria for reporting qualitative research (COREQ) [10].

### Sampling

The sample interviewed was comprised of individuals with current or previous experience overseeing or implementing anti-malarial R&D of drugs, vaccines or diagnostics in China. These individuals were located in central government agencies, pharmaceutical companies, or universities and research institutes. All of the KIs were identified through purposive sampling and snowball sampling [11]. According to the different functions of each organization, the informants were divided into three groups for purposes of data analysis, i.e., observer, administrator, and implementer. Observers were from administrative departments or non-profit organizations (e.g., universities, research institutions or international organizations), who are familiar with but do not fully participate in the R&D process. Administrators were those working in pharmaceutical companies or non-profit organizations that directly lead R&D programmes. Implementers were those who work for the administrators and participate in the R&D process.

### Data collection

The interviews were conducted by the principal investigator (YH) and by field researchers. The interviews were conducted using semi-structured interview guides. Interviews were audio recorded, transcribed verbatim and imported into QRS Nvivo Version 10, a software package for coding, organizing, management, and analysis. As part of their training, field researchers were also instructed to record notes during the interviews.

### Data analysis

Data were analysed using thematic framework analysis [12] which incorporated both deductive categories (questions from the interview guides) and inductive findings (unanticipated comments) to enrich the study findings. The principal investigator, with the assistance of note takers, read the notes and provided clarification as necessary. The field notes were used to support discussions in order to confirm that all points had been accurately understood. The narratives were read and coded by an independent researcher from Peking University; themes were then compared, discussed and agreed upon together with the principal investigator.

## Results

### Participants

Interviews were conducted with 26 key informants. A total of 9 observers, 7 administrators and 10 implementers were interviewed (Table 1). Among the observers, five were leaders from four different administrative departments, including National Health and Family Planning Commission (NHFPC), State Intellectual Property Office of the People’s Republic of China (SIPO), China Pharmaceutical Innovation and Research Development Association (PhIRDA), and China Chamber of Commerce for Import & Export of Medicines & Health Products (CCCMHPIE). Observers also included representatives from non-profit organizations, administrators and implementers from China CDC, Peking University, Fudan University, Shanghai Jiaotong University, Guangzhou University of Chinese Medicine, and Southern Medical University. Interviews were conducted with key individuals located at all of the China-based pharmaceutical companies involved in anti-malarial R&D, i.e., Guilin Pharmaceutical (Shanghai) Co Ltd (GPSC) and Artepharm Co Ltd.

**Table 1:** Number of interviews conducted by type of respondent and organization

Type of respondent	Type of organization	Number of respondents (number of organizations represented)
Observer	Administrative department	5 (4)
	Nonprofit organization	4 (4)
Administrator	Nonprofit organization	4 (3)
	Pharmaceutical companies	3 (3)
Implementer	Nonprofit organization	5 (4)
	Pharmaceutical companies	5 (3)

### Awareness of the global market and WHO PQ for Chinese anti-malarial drugs

The production of Chinese anti-malarial drugs is mostly export-oriented. All the interviewers were aware of WHO PQ and its importance for entering the global marketplace. Respondents indicated that most of the Chinese anti-malarial drugs without WHO PQ could not enter the African public market, and can only be purchased for private markets in African countries, which is a much smaller share compared to the public sector.

*‘As far as I know, more than half of the products in African private market are from China. Chinese anti*-*malarial drugs are acceptable in the private market.’* (Implementer)

*‘There are multiple ways to export* [Chinese medicine]*. In order to be adopted by foreign countries, you have to meet some prerequisites. First of all, usually this product should have passed the WHO PQ, which increases the reliability of this product. Second, this product has to register in local countries.’* (Administrator)

### Barriers to achieving WHO prequalification

#### High upfront costs and unpredictable benefits

Although most KIs from pharmaceutical companies and administrative departments indicated that WHO PQ is a prerequisite for Chinese anti-malarial drugs to enter the global public market, they all expressed limited interest in applying for WHO PQ. The KIs explained that WHO’s requirements for achieving PQ are quite different from China’s existing requirements for good clinical practices (GCP), good laboratory practices (GLP), and good manufacturing practices (GMP). Chinese manufacturers indicated they would need to invest tens of millions of dollars to upgrade their factories and to hire or train qualified professional and technical personnel in order to meet WHO PQ requirements.

Key informants further indicated that even after their product passed WHO PQ, sales and level of exports of anti-malarials would be unpredictable. KIs noted that PQ is only a first step for a Chinese medical product to enter the international public sector market. Whether international organizations or other countries choose to purchase the product from the PQ list is still difficult to predict. Since the economic benefits are not clear even after a high level of investment, few respondents indicated that they want to make such investments.

*‘Without the support of government, a single enterprise needs to invest about 20*–*30 million Yuan* [3.1–4.7 million dollars] [to apply for WHO PQ], *but the sale of the products might be less than 2 million Yuan* [0.3 million dollars]. *Why should I do this?’* (Implementer)

*‘I think that business is very practical. Enterprisers need to see that this* [passing the WHO pre-certification] *will actually bring benefit. Without more than 90% chance to earn all the investment back, enterprisers won’t start to apply* [for PQ]*… It is impossible for enterprisers to invest huge amount of money without telling them the benefit. You have to reassure enterprisers during this process: reduce risk, answer their doubts and basically promise them a successful result.’* (Administrator)

#### Limited information and technical support

In terms of applying for WHO PQ, Chinese manufacturers indicated that they believe they are experiencing an information asymmetry. That is, some KIs proposed that although they intend to apply for PQ, they do not know the specific requirements. Nor do they know how to apply. Further, a perceived lack of technical support, absence of quality control system and poor quality control were also mentioned as barriers for achieving WHO PQ. Although WHO and some of China’s relevant stakeholders have held PQ training in China, the KIs felt that the information they gained during training was not sufficient to proceed with WHO PQ applications. They suggested that targeted and continued interactions might be more useful.

‘[haven’t applied] *they don’t know it since asymmetric information. This is a process of multi*-*cooperation. We need to know not only how much we should invest, but also how to update or improve. We need technological guide and professional supports, and a plan.’* (Administrator)

### Potential approaches to overcome barriers

Based on the barriers mentioned above, some KIs from pharmaceutical companies indicated their enthusiasm for WHO PQ would be higher if some approaches could be implemented, particularly incentives related to taxes and to the drug approval process. The suggestions highlighted by the KIs are listed in Table 2.

**Table 2:** List of potential incentives for WHO PQ identified by key informants by respondent type

Incentive	Total number	Observer	Administrator	Implementer
Fast track	3	0	2	1
“1 + 1” pattern	1	0	1	0
Government collaborates with user countries	3	0	1	2
Tax relief	2	0	2	0
Human resources training and consultation	1	1	0	0

Two of the three KIs from the pharmaceutical administrator group suggested that tax relief for those pharmaceutical companies that produce anti-malarial products would be the most direct incentive for company administrators to pursue WHO PQ. Also, due to the long waiting list for obtaining new drug approval from the China Food and Drug Administration (CFDA), a Fast Track process for such medicines, or a ‘1 + 1’ Fast Track Channel would incentivize interest in WHO PQ. As the KIs explained, if a pharmaceutical manufacturer is planning to apply for WHO PQ, entering the Fast Track process would speed up the application process. While 1 + 1 Fast Track Channel means that if manufacturers apply for an anti-malarial drug, they would be able to choose another drug to enter into the Fast Track as well. They mentioned that these incentives might also be useful for other medical products that lack market incentives, such as other medicines for neglected diseases.

In addition to the above-mentioned direct incentives for pharmaceutical companies, KIs indicated that the Chinese Government could encourage companies by guaranteeing volume of purchased drug orders after achieving WHO PQ. The KIs indicated that through multilateral or bilateral negotiations, the government could promote Chinese anti-malarial drugs to be included into other countries’ drug list or procurement list, and further promote Chinese anti-malarial drugs to enter the international public sector market. One observer mentioned that providing human resource training and consultations related to WHO PQ might be critical to encourage pharmaceutical factories, and make WHO PQ more possible for companies that lack the necessary information.

## Discussion

Although WHO PQ has provided access for anti-malarial drugs to enter the global market, few Chinese pharmaceutical companies have achieved WHO PQ for anti-malarial drugs. This study used qualitative methods to understand the underlying reason behind this situation, with the intent to offer suggestions on how to accelerate progress not only for anti-malarial drugs, but potentially for other Chinese medical products to enter into global public sector market.

This study included all of the KIs that were identified as involved with R&D of anti-malarial drugs in central government agencies, pharmaceutical companies, as well as universities and research institutes. According to the interviews, most respondents recognized that the lack of WHO PQ has become a huge barrier for Chinese anti-malarial products to enter global public sector market. According to previous research, the total export value of artemisinin-derived FPPs has been increasing and has roughly equaled that of artemisinin-derived APIs from 2012 to 2014 [9]. This indicates that China is moving away from exporting raw APIs to FPPs, which are more technologically advanced and profitable. However, without WHO PQ certification, most of Chinese anti-malarial drugs are destined for the private retail market in Africa or sold through intermediaries that are exporting FPPs to a third country. Entering the African market through a third country reduces the profit for Chinese pharmaceutical manufacturers, likely limits the affordability of Chinese anti-malarial drugs in Africa, and potentially stunts R&D of Chinese medical products. Anti-malarial drugs are mostly provided through the African public sector market. Since WHO PQ is the fundamental prerequisite for most international public sector purchasers and for entering the public sector market, WHO PQ could expand the global production of Chinese anti-malarial drugs.

While the merits of achieving WHO PQ are well accepted in China, existing barriers, such as high upfront costs, unpredictable benefit, as well as limited information and technical support have slowed application for WHO PQ. Every country has its own standard for pharmaceutical production. Due to the differences between Chinese GMP, GCP, GLP and WHO requirements, few enterprises could likely pass WHO PQ without high upfront costs, such as purchase of new equipment or even building a new factory. This may be the main reason why few Chinese pharmaceutical companies start the WHO PQ process, especially when investment may not lead to predictable sales and profits.

This study indicated that some incentives might accelerate the WHO PQ progress in China, including supportive policies related to tax and the drug approval process. Since WHO PQ requires a drug production license of the local country, the slow process in China of applying for national license sometimes stops pharmaceutical factories from applying for WHO PQ. Many other countries have used similar incentives to accelerate R&D for certain medical products, such as the ‘Fast Track’ or ‘Accelerated Approval’. Also, tax relief could be a great incentive for pharmaceutical companies. This means that if a pharmaceutical company has been involved in anti-malarial production, it could have a percentage of tax relief. Such accelerating processes could be incentives for pharmaceutical companies to focus on products that mainly affect developing countries. However, government support is the foundation for making these incentives possible.

In order to facilitate the development of WHO PQ in China, more incentive policies and financial support from government should be provided. Initial funds for R&D could be provided by the Chinese Government. A successful example of collaborating with government in China is the WHO PQ-intravenous artesunate achieved by Guilin Pharmaceutical, a process that involved technical assistance from the non-profit organization Medicines for Malaria Venture. It shows that when government provides a favourable environment and necessary services for enterprise development, it is possible to take industry to the next level [13].

In addition to supporting the development of equipment and standard production process in pharmaceutical factories, human resource training related to WHO PQ is needed. To be specific, training administrators from Chinese pharmaceutical companies to understand the requirements and assessment process of WHO PQ will help them be well prepared to meet the requirements. WHO conducted multiple training on WHO PQ of medicines and vaccines in China between 2006 and 2010, and offered targeted training and consultation services to help over 400 pharmaceutical companies apply for WHO PQ between 2010 and 2012, with financial support from GF and the Bill & Melinda Gates Foundation [14]. WHO conducted PQ training workshops in 2013 and 2014 during the China Pharma Holdings Inc (CPhi) exhibition, and attended the CPhi exhibition to answer questions regarding FPP and API prequalification procedures in 2016 and 2017. With the consultation and human resource training provided by Medicines for Malaria Venture, Guilin Pharmaceutical became the first WHO PQ pharmaceutical company worldwide to produce intravenous artesunate in 2011 [13]. According to the KIs, training content might need to be more specific, such as targeting deficiencies in Chinese pharmaceutical companies, apart from a general introduction to PQ process and standard.

Governments could also facilitate progress by bilateral or multilateral collaboration and negotiation with African countries and international organizations. India is a good example, illustrating the importance of government participation. The collaboration established between the national regulatory agencies of India and the user country and WHO has facilitated prequalification of medical products by WHO in India [15]. The Indian Government has been collaborating with WHO to strengthen pharmaceuticals in India through the provision of technical support for the development of a Comprehensive Institutional Development Plan [16]. India has 12 major manufacturing facilities producing vaccines that are sold in the national and international market in 150 countries, making India a major global vaccine supplier. Nearly one-third of the prequalified vaccines, and over two-thirds of the medicines purchased through international organizations were produced in India [4]. Moreover, although India is one of the major importers of China’s anti-malarial drugs, it might be also a strong competitor for Chinese anti-malarial markets in Africa. At May 2017, 17 types of WHO-PQ reviewed medicines/FPPs are manufactured in China, including 11 types of anti-malarials, while 368 types of WHO-PQ reviewed medicine/FPPs are manufactured in India, including 21 types of anti-malarial drugs. Due to the high percentage of PQ products from India, Indian medical products have become a major brand in African countries, bringing the companies profit and the country a better international reputation.

The Chinese Government has prioritized supporting African local production of medicines, and has donated many medical products to African countries. Many Chinese pharmaceutical companies have gained certification from a certain country or a region to sale their medical products. However, while these methods could bring Chinese drugs to other countries and might seem to be a bypass for WHO PQ, in the long run, they may benefit only a few countries or regions. As mentioned by the KIs, if the Chinese Government could help Chinese anti-malarial drugs to be included in the procurement list of international buyers through multilateral or bilateral negotiation, and in some way guarantee the potential market for Chinese anti-malarial drugs, it could be an incentive for pharmaceutical companies to start the PQ process.

Strengthening collaboration with sub-Saharan African countries, and others, could be a mechanism for developing the WHO PQ process in China, such as the public–private partnership (PPP) of Chinese enterprises collaborating with scientific institutions and non-governmental organizations to obtain WHO PQ for the live-attenuated Japanese encephalitis vaccine in 2013 [17]. PPPs can share the risk and develop innovative, long-term relationships between public and private sectors [18]. However, PPPs have been somewhat controversial, in that private investors may seek to acquire a higher rate of return than the Chinese Government’s bond rate, while much of the income risk associated with a project may be borne by the public sector [19]. Contract management is a vital factor in the success of PPP collaboration.

While this study interviewed KIs from multiple representative backgrounds, it has some limitations. First, since the study was designed to understand the underlying reason for limited PQ application in China, only Chinese KIs relevant to anti-malarial R&D and WHO PQ were included. KIs from WHO or donor institutions who might have provided useful suggestions from different perspectives were not included. Second, since the KIs were located in various geographical locations, it was not possible to use focus group discussions for this study. However, conducting the KI interviews in their workplaces allowed for considerable insight into the perceived barriers and their suggestions.

## Conclusions

While WHO PQ is important and beneficial to China from a long-term perspective, KIs identified barriers to pursuing WHO PQ. Government support, as well as incentives and mechanisms for greater collaboration are likely crucial for further developing of WHO PQ anti-malarial drugs in China.

## Abbreviations

APIs: active pharmaceutical ingredients
FPPs: finished pharmaceutical products
GMP: goods manufacturing practice
GCP: good clinical practice
GLP: good laboratory practice
KIs: key informants
PPP: public–private partnership
PQ: WHO prequalification
R&D: research and development
WHO: World Health Organization

## References

[1] United Nations. Sustainable developmet goal 3. 2016. https://sustainabledevelopment.un.org/sdg3. Accessed 24 Jan 2018.

[2] WHO. Prequalification programme. Geneva: World Health Organization; 2015. http://apps.who.int/prequal/. Accessed 1 Jan 2015.

[3] Årdal C, Røttingen JA (2015). An open source business model for malaria. PLoS ONE.

[4] WHO (2018). List of prequalified medicines.

[5] WHO. Benefits of WHO Prequalification. Geneva: World Health Organization; 2011. https://extranet.who.int/prequal/content/benefits-who-prequalification. Accessed 24 July 2017.

[6] Bliss K, Boynton XL, Cha V, Chand S, Conley HA, Cooke JG (2010). Key players in global health.

[7] The People’s Republic of China. China’s Foreign Aid (2014) Information Office of the State Council. 2014. http://reliefweb.int/report/china/chinas-foreign-aid. Accessed 24 July 2017.

[8] Tu YY (2011). The discovery of artemisinin (qinghaosu) and gifts from Chinese medicine. Nat Med.

[9] Huang Y, Li H, Peng D, Wang Y, Ren Q, Guo Y (2016). The production and exportation of artemisinin-derived drugs in China: current status and existing challenges. Malar J.

[10] Tong A, Sainsbury P, Craig J (2007). Consolidated criteria for reporting qualitative research (COREQ): a 32-item checklist for interviews and focus groups. Int J Qual Health Care.

[11] Goodman LA (1961). Snowball sampling. Ann Math Stat.

[12] Srivastava A, Thomson SB, Barnett-Page E, Thomas J, Carroll C, Booth A (2009). Framework analysis: a qualitative methodology for applied policy research. JOAAG.

[13] Guilin Pharmaceutical C. The world’s first producer of WHO prequalified artesunate for injection for severe malaria. 2011. https://www.mmv.org/newsroom/interviews/world-s-first-producer-who-prequalified-artesunate-injection-severe-malaria. Accessed 24 July 2017.

[14] Huang B, Xu M, Bai D, Wu Z, Wu CF (2014). The WHO prequalification programme on medicines promotes the internationalization of pharmaceutical manufacturers in China. Chinese J Pharm.

[15] Dellepiane N, Akanmori BD, Gairola S, Jadhav SS, Parker C, Rodriguez C (2015). Regulatory pathways that facilitated timely registration of a new group a meningococcal conjugate vaccine for Africa’s meningitis belt countries. Clin Infect Dis.

[16] WHO (2017). Strengthening India’s vaccine regulatory authority.

[17] Xu M, Liang Z, Xu Y, Wang J (2015). Chinese vaccine products go global: vaccine development and quality control. J Exp Rev Vaccines.

[18] Hodge GA, Greve C (2016). On public–private partnership performance: a contemporary review. Public Work Manag Policy.

[19] Barlow J, Roehrich JK, Wright S (2010). De facto privatization or a renewed role for the EU? Paying for Europe’s healthcare infrastructure in a recession. J R Soc Med.

